# Self-Regulation of Seat of Attention Into Various Attentional Stances Facilitates Access to Cognitive and Emotional Resources: An EEG Study

**DOI:** 10.3389/fpsyg.2022.810780

**Published:** 2022-02-24

**Authors:** Glenn Hartelius, Lora T. Likova, Christopher W. Tyler

**Affiliations:** ^1^California Institute of Integral Studies, San Francisco, CA, United States; ^2^Smith-Kettlewell Eye Research Institute, San Francisco, CA, United States

**Keywords:** seat of attention, attentional stance, target of attention, body-relative space, EEG, egocenter, self-location, self-regulation

## Abstract

This study provides evidence supporting the operation of a novel cognitive process of a somatic seat of attention, or ego-center, whose somatic location is under voluntary control and that provides access to differential emotional resources. Attention has typically been studied in terms of what it is directed toward, but it can also be associated with a localized representation in the body image that is experienced as the source or seat of attention—an aspect that has previously only been studied by subjective techniques. Published studies of this phenomenon under terms such as egocenter or self-location suggest that the seat of attention can be situated in various ways within the experienced body, resulting in what are here referred to as different attentional stances. These studies also provide evidence that changes in attentional stance are associated with differences in cognitive skill, emotional temperament, self-construal, and social and moral attitudes, as well as with access to certain states of consciousness. In the present study, EEG results from multiple trials of each of 11 specific attentional stances confirmed that patterns of neural activity associated with the voluntarily control of attentional stances can be reliably measured, providing evidence for a differential neural substrate underlying the subjective location of the seat of attention. Additionally, brain activation patterns for the attentional stances showed strong correlations with EEG signatures associated with specific positive emotional states and with arousal, confirming that differential locations of the seat of attention can be objectively associated with different emotion states, as implied in previous literature. The ability to directly manage the seat of attention into various attentional stances holds substantial potential for facilitating access to specific cognitive and emotional resources in a new way.

## Introduction

Historically, the sense that there is a beam of attention emanating from oneself to the object of attention (such as a chair) was so strong in the classical era that philosophers understood it as a beam of light emanating from the eyes and reflecting back information about the object of regard. It is explicit that this beam of light/attention can be directed at will toward any object in the field of view, as is the subject of numerous attentional experiments in the modern era. Of course, such concepts are all metaphorical imagery for neural excitation processes in the brain, with a target in the neural representation of the location of objects in space, that is enhanced by a directed neural process under the voluntary control of the location of interest.

This kind of focal attention has typically been studied in terms of what it is directed toward, but the hypothesis for the present study is that this beam is also perceived to have an adjustable source in the body from which it emanates, like a fireman holding their hose over the shoulder or down by the waist. The body location from which attention is sensed to project—typically either the head or upper torso—has been the subject of a small number of studies under terms such as *egocenter* or *self-location*, without reference to its possible importance for the study of attention. This study describes the body region where attention is directed from as the *seat of attention*, to distinguish it from what attention is directed toward, which is the *target of attention*. Just as one's perspective on objects in the environment changes while walking through a forest or down a city block, a change in the seat of attention creates a shift in attentional perspective, resulting in a different *attentional stance*—a variable that has been shown to affect state of consciousness, emotional temperament, self-construal, and social and moral attitudes.

The sense of where I am located is associated in a generalized way with my overall body's location in space, but within the body some locations are more strongly associated with the felt sense of the located self (cf. James, 1890). In particular, the location from which attention seems to project is experienced as the location of the perceiving self within the body (Hartelius and Goleman, 2016). Terms that are equivalent for the current discussion such as “egocenter” and “self-location” will be used when reviewing studies that rely on those terms, while noting that this usage is not intended to evoke some notion of a complex Freudian ego or constructed self (e.g., Oyserman et al., 2012), but simply refers to the experience of *a location from which attention is directed*. For this study, “seat of attention” is the preferred term. However, all previous evidence for this psychological construct has been based on subjective report of its existence and perceived location in the intrapersonal and peri-personal body space.

In relation to this cognitive construct, the present study has three main goals:

1. To draw together published evidence in support of the subjective localizability of the seat of attention previously identified in Hartelius (2015) and Hartelius and Goleman (2016).
2. To provide objective evidence for a differential neural substrate underlying the subjective location of the identified seat of attention.
3. To determine whether differential locations of the seat of attention can be objectively associated with different emotion states, as implied in previous literature.

### Self-Location and the Egocenter

Participant reporting of experienced self-location has most commonly located the sense of self in the head (Bertossa et al., 2008), in the region of the heart, or distributed between the head and upper torso (Limanowski and Hecht, 2011; Starmans and Bloom, 2012; Alsmith and Longo, 2014; Anglin, 2014; van der Veer et al., 2018, 2019). In more objective assessments of self-location, stimuli only became associated with the self of the individual when presented near the head or upper torso, and not when presented at other body locations (Schäfer et al., 2019).

Different visually based methods have been used to determine the location of the seat of attention, described as the egocenter, and of these the method described by Howard and Templeton (1966) has been found to be the most reliable (Barbeito and Ono, 1979). The Howard and Templeton method asks participants to align different sets of two stimuli separated in space so that they point at themselves, with the result that lines drawn through each of these sets should intersect at the egocenter. This experimental assay has been used with visual stimuli (Howard and Templeton, 1966), auditory stimuli (Neelon et al., 2004), and kinesthetic stimuli (Shimono et al., 2001).

While such studies have claimed to identify a *visual egocenter* (Padula et al., 2015), an *auditory egocenter* (Cox, 1999), and a *kinesthetic egocenter* (Shimono et al., 2001), such sense-specific terms are problematic for two reasons. First, the attentional egocenter is almost certainly constructed with reliance on data from multiple senses and frames of reference (Bottini et al., 2001; Blanke, 2012; Alsmith et al., 2017), rather than a single sense; second, while the location of the egocenter may be inferred based on visual or auditory data, the bodily location from which the direction of any such stimuli is noticed is necessarily somatically felt (Velzeboer, 1957; Roelofs, 1959) rather than based solely on visual or auditory data. Given that alternating strabismus or physical injury can shift the egocenter (Dengis et al., 1998; Padula et al., 2015), it is also evident that the egocenter is more than an arbitrary point from which one imagines that a sense is projected (Fry, 1950). As a biologically grounded multisensory experience—whether that experience is conscious or is inferred from behavior—what is referred to as various sense-specific egocenters is more likely a supramodal experience of the seat of attention (Likova, 2012).

In studies of the egocenter, it is clear that its felt location is variable—in sighted or newly-blind individuals, tests for its location with visual and auditory stimuli yield similar results; however, in congenitally blind individuals the egocenter, as measured by a variation of the Howard and Templeton (1966) method, is located farther back in the head [Sukemiya et al. (2008); but see differing results from Likova (2012) based on an alternate method]. When only kinesthetic and tactual information is used to locate the egocenter, its location shifts slightly out of the mid-sagittal plane based on which hand is used for touching, and how far the tactual stimuli are from the body (Shimono et al., 2001), as predicted by Blumenfeld (1937), suggesting that the egocenter may be capable of dynamic movement among different locations.

There is preliminary evidence that the seat of attention can be deployed in parts of the body other than the head, and as far down as the upper torso; in a study with 10 participants that measured felt vertical self-location relative to the body in haptic and visual modes, most participants located themselves either at the level of the head (upper face), or at the level of the upper torso, with a minority of cases near the neck or lower face (Alsmith and Longo, 2014).

### Cognitive Correlates of Variations in Seat of Attention

Differences in self-location of the seat of attention correlate with differences in emotional temperament and cognitive capacities. In a series of eight studies involving 725 participants, Fetterman and Robinson (2013) found that, regardless of sex, individuals who located the sense of self in the head scored lower on all measures relating to emotion and empathy, higher on rational thinking, interpersonal coldness, disagreeableness, and more likely to solve moral dilemmas in a rational manner, than those who located it in the region of the heart. The latter group scored higher on affect intensity, caring and empathy, attraction to intimacy-related activities, attention to emotion, reliance on experiential rather than rational thinking, emotionality, emotional reactivity, were warmer interpersonally, and were more likely to solve moral dilemmas in an emotional manner. A later study further showed that heart-located individuals tend to have stronger religious beliefs (Fetterman et al., 2020). With respect to cognitive skills, head-locators had higher academic grade point averages and scored higher on simple tests of general knowledge than those who were heart-located (Fetterman and Robinson, 2013). Self-location and associated differences remained stable over a period of at least 9 months, suggesting that self-location likely reflects a stable disposition. The authors described these self-locations as metaphors, but they likely also reflect characteristic ways in which the participants situated their seat of attention—that is, their habitual attentional stance.

A second study associated a sense of self-location in the brain with an independent self-construal—that is, a belief that one's self is an independent, self-reliant individual; by contrast, sense of self-location in the heart correlated with interdependent self-construal—the sense that one's self is flexible and shaped by others and by social situations (Adam et al., 2015). Participants who located their sense of self in the brain donated more money to a charity for Alzheimer's disease, whereas those identifying self with the heart donated more to support work on coronary artery disease. Brain vs. heart self-location also influenced attitudes about abortion legislation and how death should be legally defined. If self-location reflects a habitual attentional stance, then the kind of stance one has may have implications for how selfhood is characterized and may influence attitudes about medical and moral issues.

The bodily situation of the seat of attention may also correlate with specific states of consciousness, as demonstrated by Marolt-Sender's (2014) study on attentional stances associated with flow states (Csikszentmihalyi and Csikszentmihalyi, 1990). Twenty-eight endurance athletes reported their self-location while performing the simple cognitive task of reading a news story, and again when evoking the memory of an experience of flow during prolonged exercise. The cognitive experience was primarily associated with a head-located attentional stance, while the flow state experience was mainly associated with a heart-located attentional stance.

Whether the seat of attention is located at a single point or restricted area in the body, or is situated more broadly over some somatic volume, appears to impact wellbeing; where perceived self-location becomes less distinct and expands throughout, or even beyond, the physical body—generally in the context of meditative practices—there can be a softening or dissolution of the sense of boundary between self and environment (Ataria et al., 2015; Nave et al., 2021) that is associated with decreased anxiety and tension, increased happiness, sense of harmony, and other-centered frames of reference (Dambrun, 2016; Dambrun et al., 2019; Hanley et al., 2020), but also with a decreased sense of mental agency (Nave et al., 2021).

Separate from meditative practices there are variations in the seat of attention other than head and upper torso. In a comparison of both headset and large-screen virtual reality contexts, some participants reported self-locations in the lower torso and below the torso (van der Veer et al., 2019). Unexpectedly, 17.9% of participants from the same study pointed to a self-location above the head—including about 10% of the participants who were in a large-screen immersive display that allowed them visual access to their own bodies. This effect disappeared when participants were asked to point to the location of self on a body template presented in the VR context, suggesting that while the unfamiliar setting may have affected the phenomenal experience of self-location, it did not impact self-location conceptually.

If the seat of attention can be situated in various places and configurations relative to the body as experienced, and if various of these attentional stances are associated with differences in positivity of experience, emotional temperament, self-construal, agency, social attitude, and states of mind, it is natural to ask whether attentional stance can be easily shifted—as enhancing one's access to various capacities and performance-enhancing states might be advantageous in educational, therapeutic, sports, self-care, and self-development contexts. Fetterman and Robinson's (2013) findings offer some possible light on this question as well. As noted, participants who self-located in the head were found to have higher grade-point averages and to score higher on general knowledge questions. In addition, however, when participants were asked to point the index finger of their dominant hand to a location either on the side of the head or on the side of the upper chest while answering general knowledge questions, they scored higher when pointing to the head location. The researchers explained this difference as the result of drawing attention to either the head or the heart, which may also facilitate some consequent shift in the attentional stance.

The seat of attention may apparently be shifted unconsciously (Fetterman and Robinson, 2013), and there is preliminary evidence that variations in the target of attention—that is, whether directed inward or outward—can be reliably detected by electroencephalographic (EEG) measurement (Hinterberger et al., 2014); however, until now there has not been clear evidence regarding whether the seat of attention is controllable at will, whether it can shift to locations other than the head or chest, or whether changes in attentional stance correlate with distinctive patterns of neural activity. Hartelius (2007, 2015) and Hartelius and Goleman (2016) has described a process for intentional placement and deployment of the seat of attention into various attentional postures or stances, and suggested that the resultant changes in where and how the attentional resources are felt to be located may be a variable in changing from a cognitive to an embodied state of consciousness.

Given that embodied states such as flow (Marolt-Sender, 2014) and neo-traditional approaches to mindfulness (Hartelius, 2015) are associated with increased positivity of experience, it may be that embodiment achieved by movement of the seat of attention out of the head and into the chest or abdomen (Hartelius and Goleman, 2016) may be associated with positive emotional states, even in the absence of meditative practices.

### Seat of Attention: Body-Relative vs. Euclidean Space

As part of the discussion of the seat of attention and bodily locations of various attentional stances, a question worthy of examination is the relationship between the felt experience of body-relative space (Shimono et al., 2001) and objective Euclidean space internal to the body. A lack of distinction regarding the nature of the space(s) under discussion goes back at least as far as Wells (1792), in his early description of how binocular vision functions. Wells showed that objects located in the optic axes of the two eyes—the line between a viewed object and each eye—will be seen to be located on the central axis—a line extending forward perpendicularly from the midpoint between the eyes. This identity realigns the visuomotor space as though the two eyes were in the same location. Thus, while the two optic axes exist in Euclidean space, the central axis exists only in visuomotor space as experienced through binocular view. For example, when a pencil is placed with the eraser against the bridge of the nose and held perpendicularly to the face, in space-as-experienced there appear to be two pencils, even though in Euclidean space there is only one.

The location at which the center of visual direction [Wells' (1792) common axis] is imagined to reside has been found to closely match the location of the visual egocenter [Barbeito and Ono (1979); cf. Hering's (1942) “cyclopean eye”], a fact that may have led to the conflation between Euclidean space and the space of lived experience that is represented by a term such as visual egocenter. Roelofs (1959), following Velzeboer (1957), pointed to this distinction, but his observation has not been widely followed. The visual axis that binocular vision constructs as its center of visual direction is projected into Euclidean space, but exists only in body-relative space.

The construction of body-relative space draws flexibly on multiple forms of sense perception, as demonstrated by the fact that subjects who are permitted non-informative engagement of the visual sense will perform haptic matching tasks more accurately than those who are blindfolded and must rely only on limb position for spatial orientation (Newport et al., 2002). Moreover, construction of body-relative space is supramodal, transcending dependence on any specific sensory input, as shown by brain imaging studies in congenitally blind, late-onset blind, and blindfolded sighted participants in a special memory-drawing paradigm (Likova, 2012). Furthermore, all participants in that study demonstrated that this supramodal *space-to-cortex encoding* took place in the frame of reference of the seat of attention, similarly located on the mid-sagittal plane of the head across both the blind and the sighted.

When the center of visual direction is conflated with the seat of attention, the cognitive concept of Euclidean space typically displaces other somatosensory information contributing to body-relative space—perhaps based on a bias that favors later developmental capacities over early ones. The visual sense is particularly vulnerable to such a conflation because it spans both concepts: Euclidean space can be understood as a cognitive objectification of space based on the visual sense (Paillard, 1987), whereas visual data are merely one source of input that informs the supra-modal mapping of body-relative space. The distinction between these two is nevertheless crucial to ensure that the seat of attention is not simply subsumed under the center of visual direction.

Given that the egocenter can be described as located in constructed body-relative space, a neural representation of the egocenter certainly exists within the brain, and its body-relative location would appear to be a qualitative projection into other brain areas such as the cortical representation of the body schema. That the egocenter can be situated within constructed body-relative space does not diminish its significance or impact: drawing on an example involving the target of attention rather than seat of attention, when the sense of body ownership is transferred from an individual's actual arm to an artificial counterpart, the temperature of the actual body part reduces, suggesting that changes in the neural body schema can have a direct and measurable effect on the anatomical body part (Moseley et al., 2008). In the sense of having effect not only on the body schema and on conscious experience, but likely also at bodily areas associated with its reported presence in body-relative space, the seat of attention can also be considered present at those anatomical locations.

More challenging to explain is the fact that self-location is in some instances reported to extend beyond or be located outside of the bounds of the physical body (Ataria et al., 2015; Dor-Ziderman et al., 2016; van der Veer et al., 2019; Hanley et al., 2020). However, body boundaries and peripersonal space can dynamically modify with experience (Canzoneri et al., 2013; Serino et al., 2015). Also, certain meditative practices have been observed to alter the sense of boundaries between self and non-self-accompanied by changes in the temporo-parietal junction (Dor-Ziderman et al., 2016)—the same brain area that has been associated with out-of-body experiences (De Ridder et al., 2007). Given that self-location, body boundaries, and the distinction between self and non-self all exhibit some degree of plasticity, it seems plausible that self-location can be experienced as extending beyond the usual bounds of the physical body.

### Seat of Attention and the Self

In recent decades new proposals for a psychological core self consisting of subcortical resources has been advanced from multiple quarters (e.g., Damasio, 1999; Crick and Koch, 2000; Panksepp, 2000, 2005; Panksepp and Northoff, 2009; Morsella et al., 2016; Solms and Friston, 2018; Solms, 2021; Tyler, 2021). While there is as yet little empirical confirmation of this construct, possible avenues of empirical research have been proposed. If there is a neurologically based core, as this nascent area of conceptualization proposes, it would be reasonable to consider whether a located experience of self might reflect some aspect of such a core self, and might therefore be reflected within neural activity in more consistent and substantive ways than those required for cortical representation.

Efforts to seek neural correlates of the seat of attention are also made plausible by the fact that changes in its location are reliably associated with access to and expression of various emotional and cognitive resources.

### Self-Regulation of Seat of Attention and Positive Emotions

In light of the reviewed literature, it is clear that the seat of attention—commonly described as self-location or egocenter—can be experienced as specifically located relative to the body (e.g., Bertossa et al., 2008), that it can be localized at different places in the body, and that these locations differ in degree of access to particular emotional and cognitive resources (e.g., Fetterman and Robinson, 2013). It has also been demonstrated that softening of the perceived boundary between the located self and the environment is associated with a general increase in positive emotional experience (Dambrun, 2016; Dambrun et al., 2019). Such findings make it possible to hypothesize that self-regulation of the seat of attention might provide access to targeted cognitive and emotional resources. To the best of our knowledge no studies have as yet tested this hypothesis. It is also not known whether various attentional stances can be differentiated through neuroimaging.

If self-regulation of the seat of attention facilitates access to various types of cognitive and emotional resources, then it is functioning as a novel cognitive process—one that should be detectable by some form of neuroimaging. Individual attentional stances should at a minimum demonstrate characteristic patterns of neural activity and there should be consistent differentiation between stances. If the seat of attention is mapped in a location such as S3, adjacent to the somatosensory cortex—Penfield's homunculus that reflects the body superior-to-inferior structure (Roux et al., 2018)—one might expect an increase in higher frequencies with attentional stances that are experienced as more superior relative to the body. Additionally, given that focused meditative states are associated with beta and gamma activity, while open monitoring (diffused) meditation styles are associated with increased activity in the theta band (Travis and Shear, 2010), one might expect focused and diffused attentional stances to display quite different—possibly even inverted—patterns of activation.

The current study set out to investigate the volitional self-regulation and possible neural and emotional correlates of specific attentional stances.

The main hypotheses of the study were as follows:

1. Participants would report (a) being able to move their seat of attention from one designated location within the body (attentional stance) to another; (b) being able to move their seat of attention to places other than the head and chest; and (c) being able to shift between focused and diffuse attentional stances at each location;
2. Each attentional stance will show a differential scalp activation pattern;
3. Within-participant inter-run correlations on the same attentional stance will be positive, indicating consistency in brain activity associated with each attentional stance;
4. EEG signatures associated with attentional stances will show an increase in higher frequencies as the seat of attention is situated higher along the body's superior-inferior axis or between focused and diffused stances;
5. The signature of some attentional stances will correlate significantly with those of positive emotions and/or affective dimensions;
6. Attentional stances that soften the sense of boundaries or extend beyond the boundaries of the physical body will be associated with positive emotional states;
7. Attentional stances that do not extend beyond the boundaries of the physical body will not be associated with positive emotional states;
8. Only focused attentional stances aligned with the body midline will be strongly correlated with emotion-related arousal;
9. Diffuse and focused stances will display inverted correlational relationships to positive emotions and arousal.

## Materials and Methods

Eight participants (5F/3M) with a mean age of 54 (range: 32–71) who reported facility in self-regulation of their attentional stance were measured with a 128-lead EEG array while assuming 11 specific attentional stances. The sample size is modest due to the difficulty of identifying individuals capable of rapidly moving through multiple attentional stances, which is rather more demanding than the common use of self-regulation for simplified access to meditative, embodied, or performance-related states. Experimental procedures were reviewed and approved by the California Institute of Integral Studies Human Research and Review Committee, and participants provided written informed consent.

Some stances required the seat of attention to be focused in a particular body location, while in others it was required to be diffused over an extended region of body-relative space. Participants completed three 20 s trials of each attentional stance. The 127-channel EEG was recorded continuously throughout the experimental session (see below). The trial protocol was identical for each participant, except that each set of stances arranged in a different randomized sequence to eliminate order effects. Participants were instructed to assume one attentional stance at a time for 20 s, asked to indicate when each attentional stance had been achieved, marking the time at which the 20 s sample was extracted from the continuously-recorded EEG.

**Figure 1:**
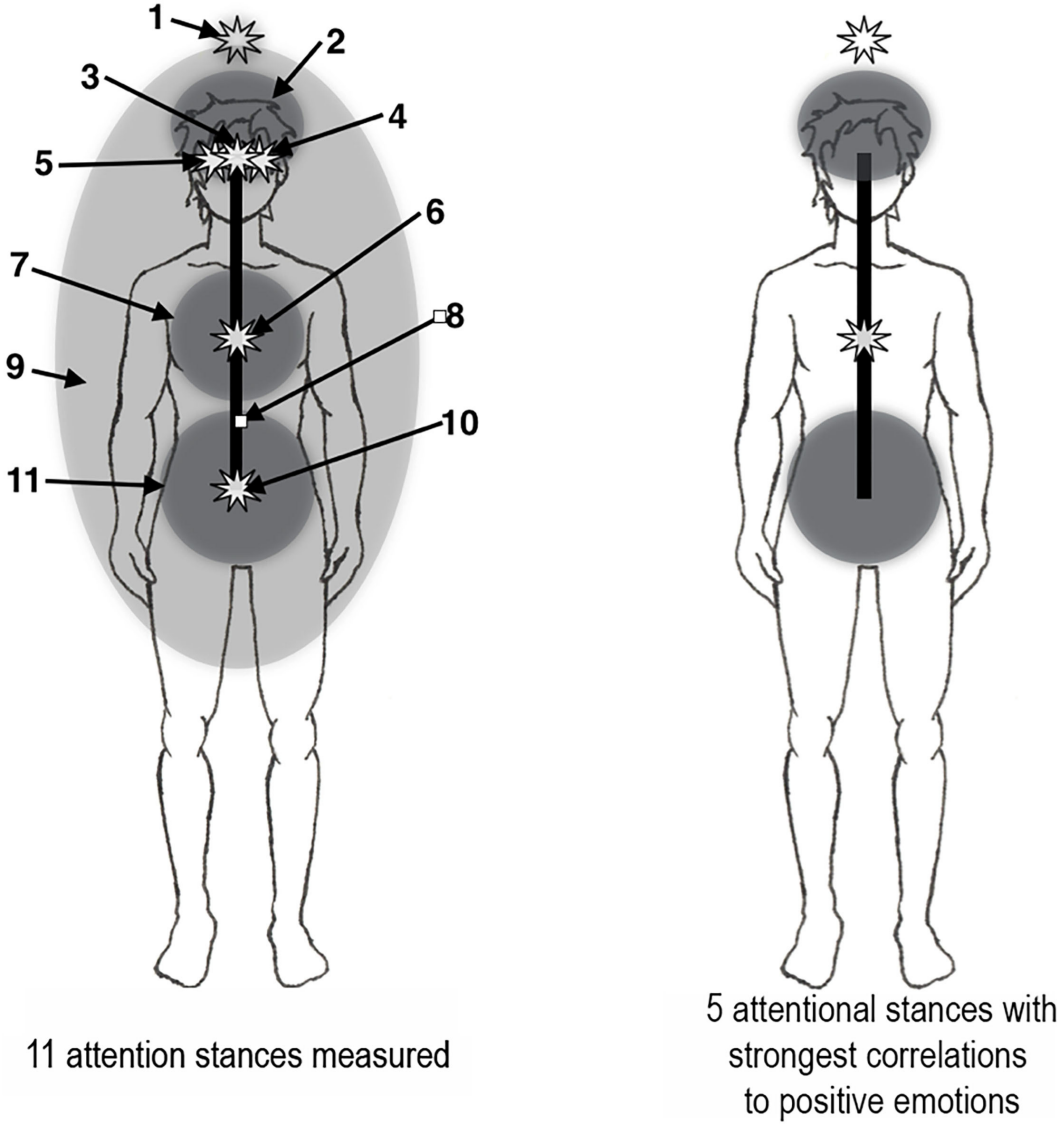
Seat of attention situated in attentional stances. Left: The 11 attentional stances measured (see text). Right: The 5 attentional stances with strongest correlations to positive emotions (see Table 1).

Normally, most people in the schooled Western culture think of their attention as emanating from somewhere in the middle of the head, but different social contexts may move it to different locations in the body. For example, when dancing or listening to rock music, the seat of attention may naturally shift to somewhere in the abdomen. Experience with individuals interested in controlling their attentional stance shows that they can report moving the seat of attention to a wide range of bodily locations, and also of controlling whether it is locally focused or more broadly diffused. Inspired by a small number of published reports of self-location extending beyond the physical body (e.g., Ataria et al., 2015; Hanley et al., 2020; Nave et al., 2021), and specifically above the head (van der Veer et al., 2019), a diffuse attentional stance extending beyond the physical body and a focused attentional stance located a short distance above the head were included. The 11 attentional stances employed in this study were as follows (Figure 1):

1. Focused at a point 10–15 cm above the crown of the head.
2. Diffused in the upper head.
3. Focused in the center of the head behind the eyes.
4. Focused in a point 3–4 cm to the left of the center of the head.
5. Focused in a point 3–4 cm to the right of the center of the head.
6. Focused in the center of the chest.
7. Diffused in the center of the chest.
8. Focused in the core of the torso and head in front of the spine.
9. Diffused in the area of the torso and head and extending about 50 cm beyond the body.
10. Focused in front of the spine at the level of the low abdomen.
11. Diffused in the low abdomen.

The attentional stances used were derived in part from the relevant literature, which regularly described head-located and heart-located self-locations; martial arts literature also makes reference to a point in the center of the abdomen reported to be of importance in praxis (e.g., Aihua et al., 2020; Cibotaru, 2021). Each of these three locations—head, heart, and abdomen—was iterated in focused and diffuse forms. Locations a few centimeters to the left and right of the center of the head were added to test for possible effects of stances located off of the body's centerline as well as any possible contralateral effects. Self-location above the head was included based on a report by van der Veer et al. (2019). Focused and diffuse stances centered on the core of the body were added as logical aggregations of head, heart, and abdominal stances. The strategy used for navigating to different attentional stances was described by Hartelius (2007). No assessment of emotional states was made.

### EEG Recording and Analysis

**Figure 2:**
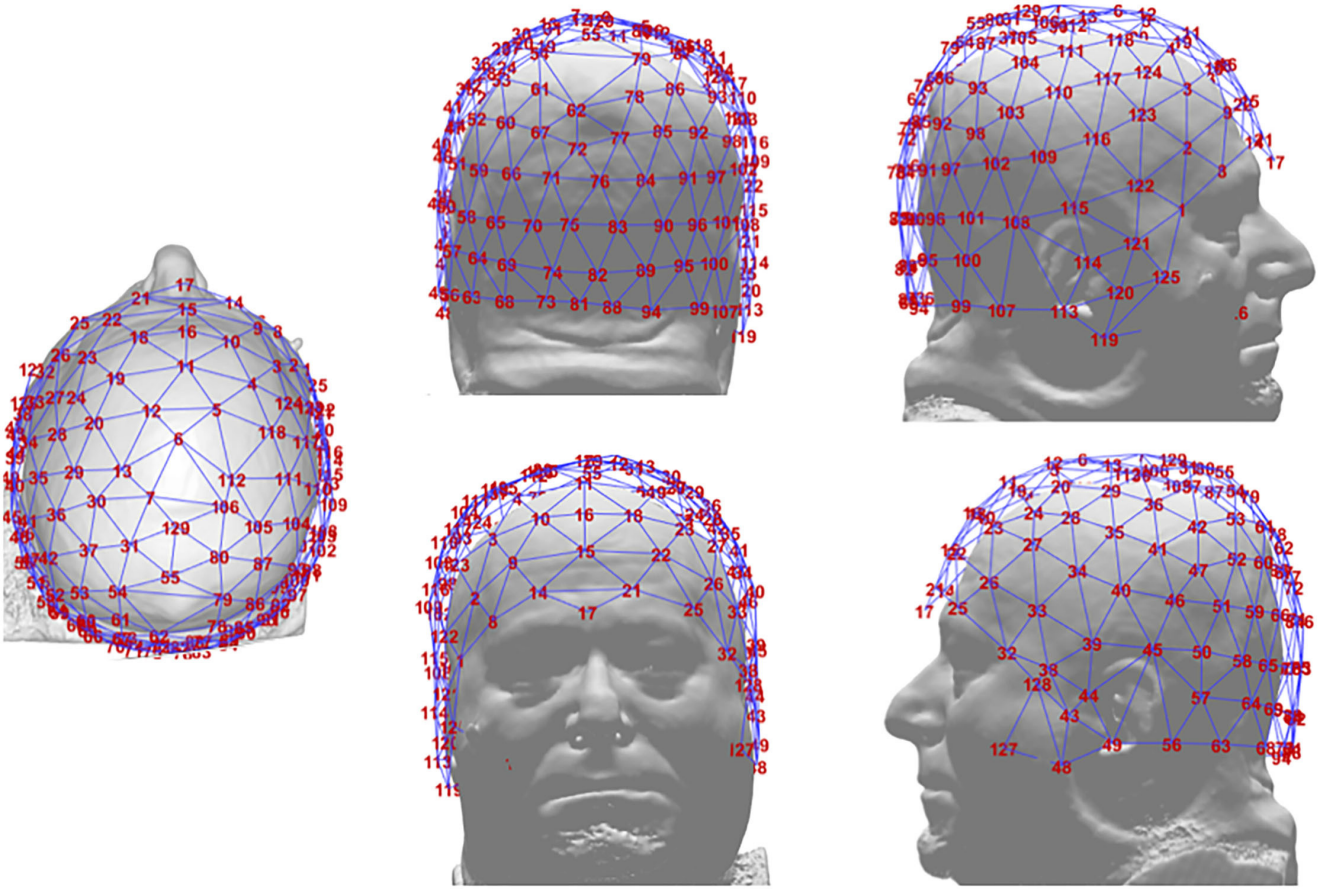
Configuration of the Netstation 128-electrode net from five viewpoints on an example head.

The EEG was recorded continuously throughout the experimental session and the 20s epoch corresponding to each attention stance event extracted on the basis of a trigger signal recorded when the participant indicated that the designated stance had been achieved. EEG data were recorded using an EGI Geodesic Netstation high-density, whole-head recording system (Electrical Geodesics, Inc., Eugene, OR), which incorporates 128 electrodes distributed around the head and face, so that the electrode net spans all regions of the scalp overlying any part of the brain (Figure 2). The impedance of all electrodes was maintained below 60 kΩ according to the recommended value for this system. Data were recorded at a sampling rate of 500 Hz with a pass-band from 0.01 and 250 Hz. The recorded signals for all electrodes were referenced to the vertex electrode (Cz).

**Figure 3:**
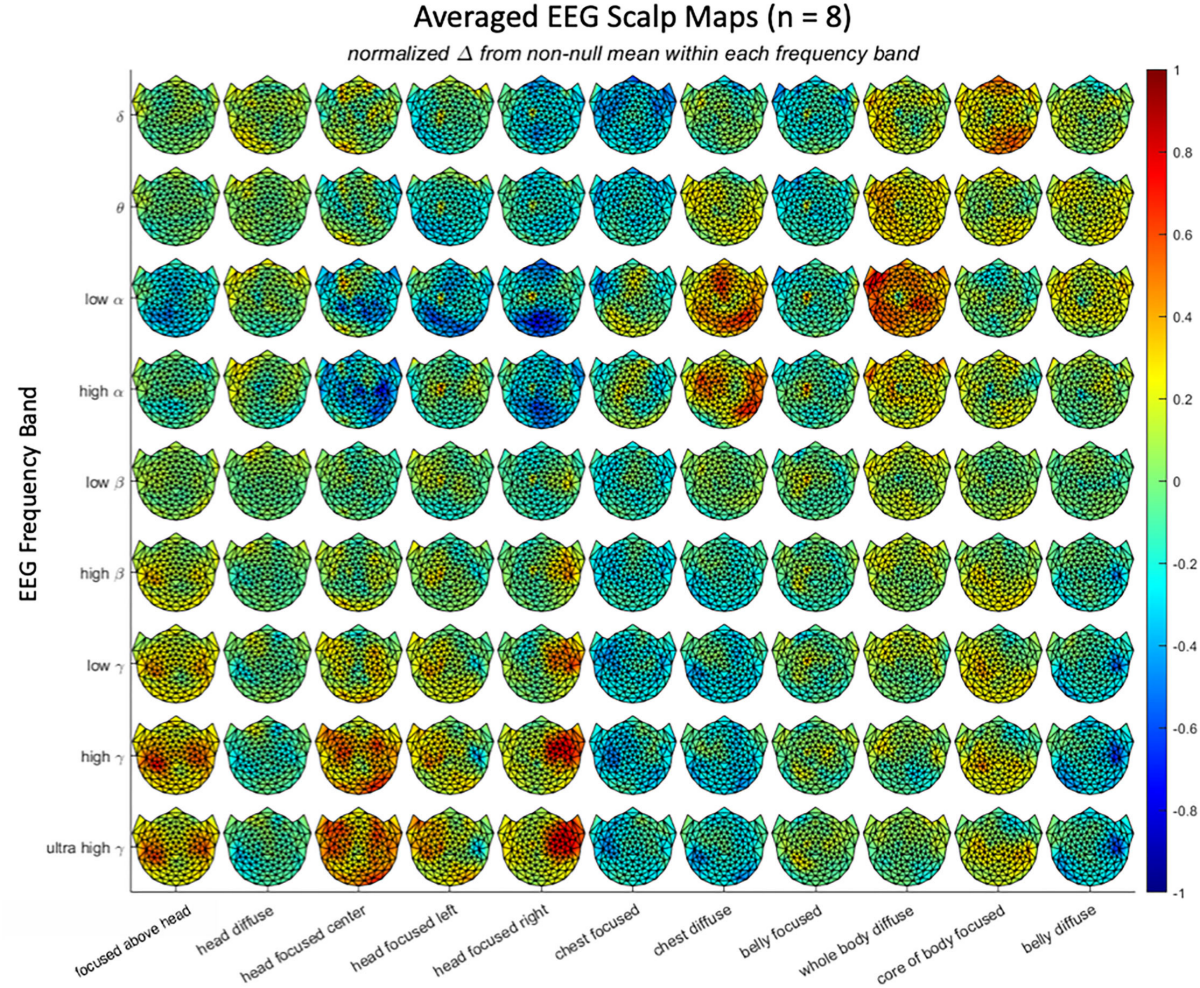
Average scalp maps of activation in the 11 attentional states for each of 9 EEG frequency bands, relative to the null state of reading text. Color bar shows the coding for normalized EEG amplitude from negative to positive.

EEG results were analyzed by a normalized frequency-band analysis that was validated by a frequency-domain principal components analysis. One approach to frequency-based analysis is to apply a principal components analysis (PCA) to the frequency spectra across conditions to identify the primary components controlling the differential EEG activity during the attentional tasks. For this, the data were first filtered with a filter proportional to frequency (f), because EEG spectra tend to fall with a 1/f spectrum up to about 100 Hz, while the signal-to-noise ratio tends to remain roughly constant over the same range. The PCA was then computed by the Matlab Singular-Value Decomposition (SVD) function. The eight components contributing the most to the PCA variance are plotted in Figure 3. They show a number of notable narrowband peaks, supporting the general approach to EEG analysis and indicating variation across the attentional stances in the following frequency bands: delta (1–4 Hz), theta (4–7 Hz), low alpha (8–11 Hz), high alpha (12–14 Hz), beta (15–30 Hz), gamma (31–100 Hz).

Inter-run correlations of the EEG frequency spectrum amplitudes were computed, for each combination of frequency band and attentional state, across the three runs for each participant. Blink artifacts were avoided by subdividing the runs into 2s epochs and restricting the inter-run correlation analysis to samples in which the epochs were blink-free.

The EEG scalp patterns of 10 positive emotions and 4 related affective dimensions were measured by Hu et al. (2017) while watching a set of emotionally-arousing movie sequences that had been previously scored for these emotions by an independent panel of raters on 7-point Likert scales. The 10 positive emotions were Amusement, Awe, Gratitude, Hope, Inspiration, Interest, Joy, Love, Pride, and Serenity. The four affective dimensions were: Arousal, Valence. Familiarity, and Liking. The data for these scalp patterns were kindly provided by those authors for the purpose of comparison with the present data on attentional stances, which were downsampled from the 127 electrode locations for best match to their 32 locations.

For the emotional analysis, the patterns of activation over these 32 scalp locations for each of 10 emotions and 4 affective dimensions were correlated with the patterns of activation across these locations in the present study, for each of the 11 attentional stances in each of the 5 EEG frequency band (a 14 × 11 × 5 correlation matrix).

### Statistical Analysis

The approach taken to the group-level statistical analysis is to rely on individual significance levels for each correlation of the pattern of scalp activation for each of the 10 emotions with that in each frequency band for each of the 11 attentional stances, with correction for multiple applications. Across 32 scalp locations, this correction provides for correlations ≥0.5 being treated as significant at *p* ≤ 0.002, a level that would allow less than one false positive per frequency band.

## Results

In lieu of unwieldy ANOVA statistics, results are only reported if they exceeded a criterion of 3 standard errors of the mean (corresponding to *p* < 0.001, or Bonferroni criterion of *p* < 0.05 corrected for 50 applications of the test). This approach combines statistical rigor with a flexible analysis capability for complex data structures. Given the sample of 8 participants, this criterion corresponds to an effect size of 1.06 for any reported result, which is well beyond Cohen's (1992) criterion of 0.8 for a robust effect size.

As anticipated, participants self-reported the ability to shift from one attentional stance to another with relative ease (Hypothesis 1a). Transitions between attentional stances typically required <1 min to achieve as indicated by the participants with a slight nod of the head following the instruction about which state to target at the beginning of each trial, including some seconds allowed for stabilization of the stance. All participants reported the ability to shift to each of the 11 different attentional stances prescribed for the study, including locations in the lower torso (Hypothesis 1b), and were able to enter both focused and diffuse attentional stances (Hypothesis 1c).

**Figure 4:**
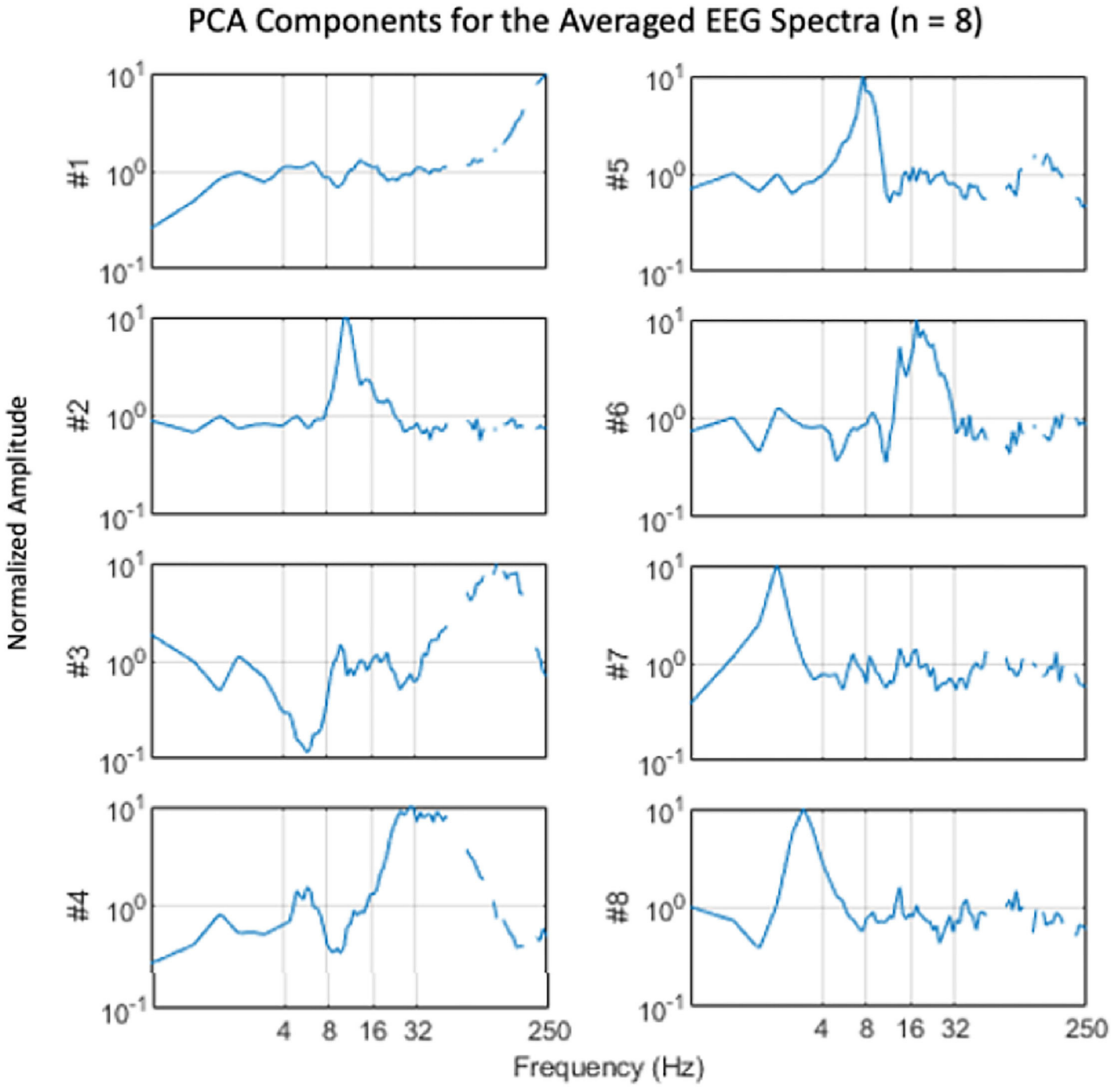
Average spectral components derived by principal components analysis. Note the tendency for components to be focused on narrow frequency bands at 2 (#7), 3 (#8), 8 (#5), 11 (#2), 18 (#6), 25–40 (#4), and 120–160 (#3) Hz (Gaps at higher frequencies are spectral regions excluded from analysis due to electrical interference).

Each attentional stance showed a differential pattern of scalp activation in at least one frequency band (Hypothesis 2); the average responses across the three repeats for all 127 active electrodes under the 11 attentional stances in each of 9 EEG frequency bands are shown in Figure 4. Note that there is a wide range of scalp patterns, including a unique pattern for most of the attentional stances in one or more frequency bands. At the same time, there are similarities in the scalp patterns for vertically adjacent frequency bands—particularly in the higher frequencies—suggesting that it would be appropriate to compress them to fewer frequency bands.

**Figure 5:**
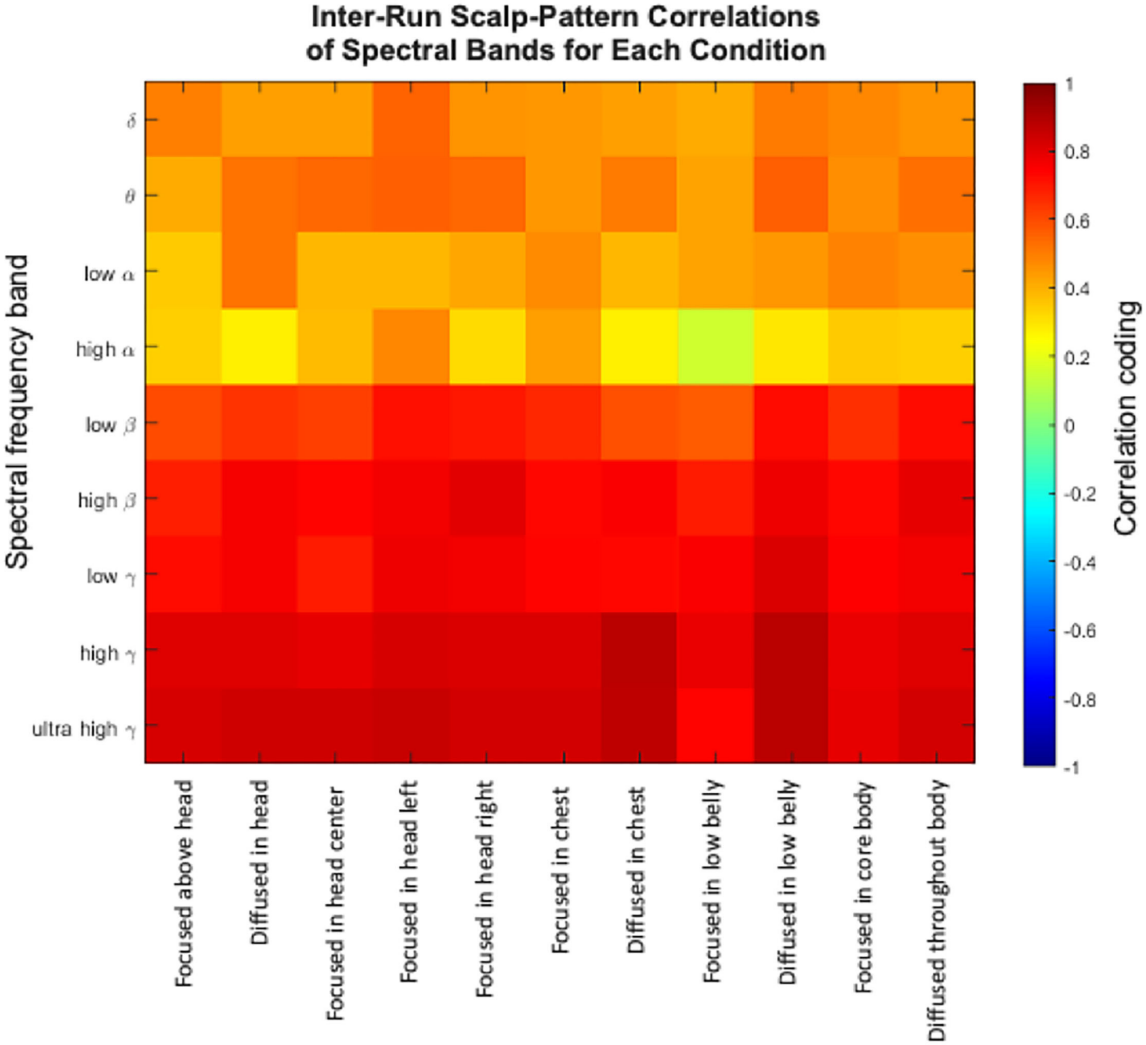
Average inter-run correlations for eight participants.

Multiple analytic approaches found no discernible superior-to-inferior or focused-to-diffused pattern across the EEG frequencies (Hypothesis 3), suggesting that the seat of attention is encoded elsewhere than near the somatosensory cortex. Moreover, within-participant inter-run correlations for each attentional stance were positive for all measured frequency bands. Inter-run correlations were lowest in the alpha bands but higher than 0.5 in all bands above the alpha frequencies (Figure 5; Hypothesis 4).

Largely in line with expectations (Hypothesis 6), four of the five attentional stances expected to soften the sense of boundaries through diffusion or extension beyond the bounds of the physical body were strongly correlated (≥0.5) with at least one positive emotion in one band (focused above head, head diffuse, chest diffuse, abdomen diffuse, whole body diffuse). Only two of six attentional stances focused within the bounds of the physical body were associated with positive emotions, which was partially in line with predictions (Hypothesis 7; chest focused, core of body focused). As predicted (Hypothesis 8), the three attentional stances associated with arousal were aligned with the body midline (focused above head, focused in center of head, focused in core of body).

In three of the four conditions where a similar seat of attention was used for both a focused and diffuse attentional stance (head, abdomen, whole body), the focused and diffused conditions correlated with positive emotions in an inverse way in the beta band (**Figure 7**; Hypothesis 9); this was also the case in the gamma band for two of these conditions (abdomen, whole body). For example, when attention was focused at the core of the whole body, beta activity correlated positively with four emotions (amusement, interest, joy, love) and negatively with six emotions (awe, gratitude, hope, inspiration, pride, serenity); when attention was diffuse throughout the whole body, beta activity correlated in precisely the opposite way—negatively with the four emotions that showed positive correlations with the focused state, and positively with the six emotions that had negative correlations in the focused state.

A similar inverse relationship between focused and diffuse attentional stances was observed relative to arousal (**Figure 7**; Hypothesis 9). In three of the four locations of attention where both focused and diffuse stances were used, the correlation with emotion-related arousal was inverted. That is, in the beta and gamma bands three focused attentional stances (head, abdomen, whole body) correlated positively with arousal levels associated with positive emotions, while for three corresponding diffuse attentional stances (head, abdomen, whole body) correlations with arousal were negative to approximately the same degree.

With the exception of the chest location, the center-line focused attentional stances show similar profiles of positive and negative correlations across ten positive emotions and arousal (above head, head, abdomen, whole body). This is also the case with the diffuse attentional stances (head, abdomen, whole body), except that as noted the profiles of diffused attentional stances are inversions of those for the focused attentional stances.

Given that there were no such inverse correlations in theta, low alpha, or high alpha bands, and that focused and diffuse attentional stances situated in the chest did not show the predicted inverse correlations, Hypothesis 9 was only partly confirmed.

### Correlations With Positive Emotional States

**Figure 6:**
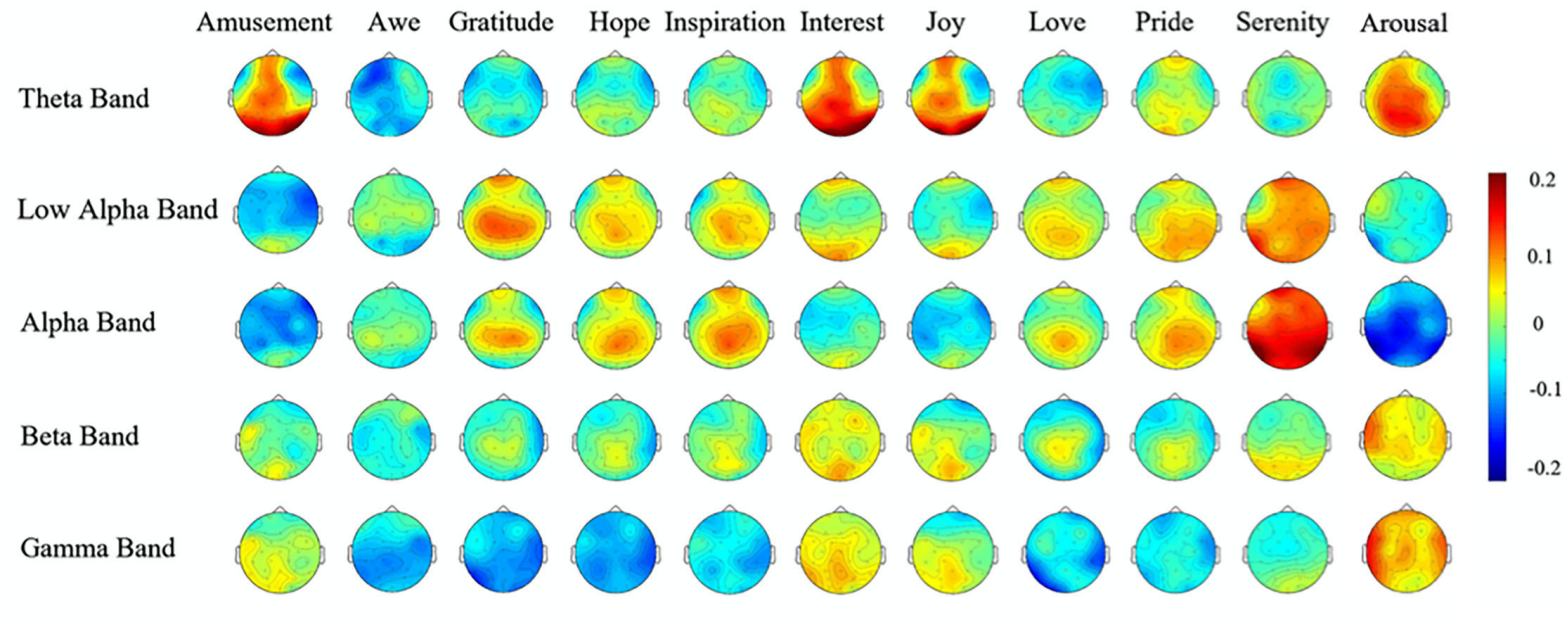
EEG spatial patterns for 10 positive emotions, plus arousal. Note that these emotions are a mostly highly distinct from each other in one or more frequency bands [from Hu et al. (2017); reproduced with permission].

**Figure 7:**
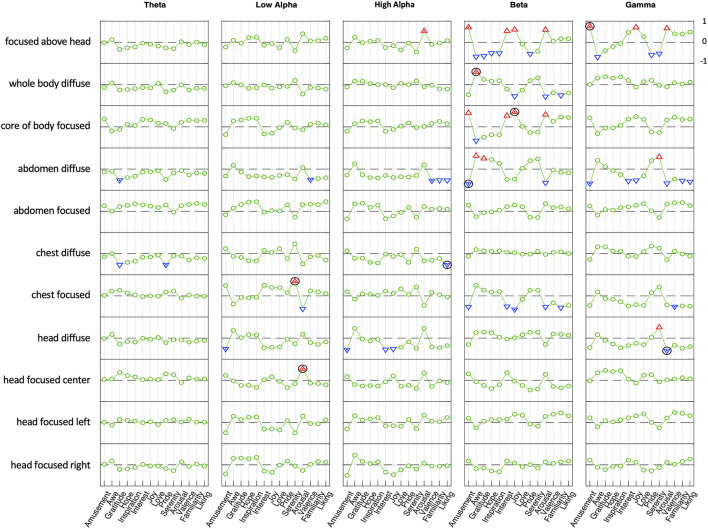
Correlation between average power spectra (differences from non-Null mean) and emotional correlation topography in Hu et al. (2017) in five frequency bands. The abs[correlation] ≥ 0.5 are indicated by triangles. Across 32 scalp locations, correlations ≥ 0.5 are significant at *p* ≤ 0.002. The maximum within-band abs[correlation] above this threshold is marked with a dot. The frequency band with maximum correlation for each state is marked with a black circle.

Of the 11 attentional stances, six showed strong positive correlations (≥0.5) in at least one frequency band with the EEG signatures reported by Hu et al. (2017; see Figure 6) associated with positive emotional states or with arousal, as shown in the correlations across the 32 electrodes of that study with the activation patterns of the present study, downsampled to 32 spatial samples (Figure 7 and Table 1,). This result validates hypothesis 5, that the patterns of attentional EEG activation would correlate with the patterns of the positive emotional states and/or affective dimensions. The highest significant correlation in each attentional condition is of the order of 0.8, which is significant at *p* < 0.0001 for the 31 degrees of freedom of the scalp locations, implying no false positives over the 770 tests conducted for Figure 7. There were also a similar number of negative correlations, many in the same frequency bands as the positive ones. Indeed, all 14 of the Hu et al. variables are significantly correlated with at least one attentional stance in some frequency band.

**Table 1:** Band-specific correlations between attentional stances and positive emotions or arousal ≥ 0.5 (i.e., significant at *p* ≤ 0.002).

**Band** **attentional stance**	**Theta**	**Low alpha**	**Alpha**	**Beta**	**Gamma**
Above head focused			Arousal	Amusement Interest Joy Arousal	Amusement Joy Arousal
Head diffuse					Serenity
Head focused C		Arousal			
Head focused L					
Head focused R					
Chest focused		Serenity			
Chest diffuse					
Core focused				Amusement Interest Joy Arousal	
Whole body diffuse				Awe	
Abdomen focused					
Abdomen diffuse				Awe Gratitude	Serenity

The specific positive emotions that were found to show a pattern correlation with the attentional stance patterns are tabulated in Table 1. Nearly all correlations occurred in the higher frequency bands, which tended to correspond with the more active emotions. In particular, four positive emotions were associated with two attentional stances in the beta band: Amusements, Interest, Joy, and Arousal, with most of the other emotions showing negative correlations in these conditions. The two attentional stances showing this characterization were the Core of Body Focused and Focused Above Head stances. The corresponding diffuse stances, Whole Body Diffuse and Abdomen Diffuse tended to show the opposite pattern of correlations.

## Discussion

Selective attention is well-known to be directable to specific targets in the field of awareness, both exogenously and under voluntary control. What has not been previously established is whether the *source* of this attentional focus, the felt location of the perceiving self, is always at the same bodily location (such as the center of the head), or whether its felt location can be relocated under voluntary control. This study had two major goals. The first was to provide objective evidence in support of the concept that there is an identifiable seat of attention located somewhere in intrapersonal space that can be voluntarily controlled. The second was to utilize the pattern of EEG activation associated with each attentional seat, or stance, included in the study to identify the emotional tone accompanying each one.

The results offer the first evidence that the self-regulation of this seat of attention in the felt experience of body-relative space can be used to voluntarily access specific attentional stances, and that these stances can be situated in areas of the body other than the head and chest. These attentional stances were reliably associated with specific patterns of brain activation, and activation patterns differed between stances. Attentional stances therefore appear to correlate with stable patterns of neural activity. This suggests that the construction and regulation of the seat of attention is a novel cognitive process not previously assessed.

The fact that regulation of the seat of attention appears to directly affect access to emotional and cognitive resources is in line with previous findings that the subjective experience of self-location served this function (Fetterman and Robinson, 2013; Adam et al., 2015; Fetterman et al., 2020). Given that the seat of attention reflects an experience of self based on a stable set of variable neural correlates, these results may also be early empirical evidence of what has been proposed as a semiconscious core self (Damasio, 1999; Panksepp, 2000, 2005; Panksepp and Northoff, 2009; Morsella et al., 2016; Solms and Friston, 2018; Solms, 2021). While EEG measures cortical activity, a core self-based in subcortical resources would be robustly interconnected with higher resources and likely also reflected in activity of the cortex.

Six of eleven attentional stances were strongly correlated with one or more of six positive emotions (gratitude, serenity, joy, awe, amusement, and interest) in at least one frequency band. This suggests that selection and use of attentional stances associated with different cognitive and emotional resources may offer a simplified way to access desirable mental and emotional states and their associated resources. Since participants in this study were typically able to relocate attention from one stance to another in less than a minute, self-regulation of the seat of attention may provide a rapid and efficient way to access such resources.

Applications of the findings will require additional research to clarify the mechanisms by which self-regulation of the seat of attention impacts cognitive, emotional, and attentional states. However, in order to guide the development of future hypotheses it may be useful to consider ways in which attentional stances may be related to various mental states.

For example, typically complex or high-performance states such as mindfulness or flow states are accessed by indirect means and learned slowly. Training in state mindfulness in a mindfulness-based stress reduction context, while effective, will often require 25–30 h of instruction plus hours of individual practice before the target state can be reliably accessed and retained, and the skill maintained. This is likely in part because the mindful state is taught through inductive learning, as a byproduct of repeated exercises in a group context where a leader is modeling the mindful state. By contrast, training based on the attentional stance of the mindfulness state could provide greatly accelerated learning and expedited access to such states.

As a specific example, in Marolt-Sender's (2014) study with 28 endurance athletes who reported having flow experiences during exercise events, the first author led a 3-h intervention in which participants were taught how to move their seat of attention into a variety of attentional stances. After learning how to self-regulate their seat of attention a number of participants commented informally that they realized they could simply access a flow state directly rather than waiting for it to occur during sustained athletic activities.

Self-regulation of the seat of attention to head or chest locations may also provide direct access to cognitive and affective states associated with head-located or chest-located attentional stances. Evidence from prior work suggests that the seat of attention, corresponding with self-location, may be a variable in cognitive efficacy, emotional temperament, self-construal, and social and moral attitudes (Fetterman and Robinson, 2013; Adam et al., 2015; Fetterman et al., 2020), and that directing attention toward a particular self-location may enhance the associated resources (Fetterman and Robinson, 2013). Learning how to self-regulate the seat of attention to a head-located attentional stance for cognitive activities and to a chest-located stance for emotional contexts may in this way serve to bring forward or enhance the personal resources appropriate to a given situation.

Furthermore, initial studies have suggested that meditative practices that soften the sense of self-boundaries are associated with increased happiness, a sense of harmony, and decreased anxiety (Dambrun, 2016; Dambrun et al., 2019). This accords generally with evidence from the current study in which four of the five attentional stances associated with positive emotions in the higher frequency bands associated with active emotions were either diffuse or situated beyond the bounds of the physical body (head diffuse, abdomen diffuse, whole body diffuse, focused above head). The attentional stance located at the core of the body was also associated with positive emotions, and though focused was felt as extending vertically through multiple areas of the body.

It may be that just as visual focus enables distinctions through suppression of the visual ground (Likova and Tyler, 2008), focused attentional stances heighten the self/environment distinction in a way that is comparable to visual processing of figure/ground, where self becomes the highlighted figure and environment is the ground that is suppressed. Conversely, attentional stances that are diffuse, extended, or situated beyond the boundaries of the physical body may serve to soften the self/environment boundary in ways similar to meditative exercises such as the body scan; as noted, practice of “body scan” meditation has been associated with increased happiness and sense of harmony, and decreased anxiety (Dambrun, 2016; Dambrun et al., 2019).

Conceptualizing of the seat of attention and attentional stances may also be of use in making more practical descriptions of various meditative states. Meditation practices have long been divided between at least two basic types: concentrative or focused attention, and open monitoring or distributed attention (e.g., Goleman, 1972; Ainsworth et al., 2013). However, these categories—which are based on characterizations of the target of attention rather than the seat of attention—have proven inadequate to explain evidence that meditation practices of both types from the Theravada tradition of Buddhism had calming effects, whereas practices of both types from Vajrayana Buddhism resulted in arousal (Amihai and Kozhevnikov, 2014, 2015).

In light of this preliminary evidence, the seat of attention—that is, where attention is felt to come from in the body—may be as important as how it is directed. The ability to correlate discrete variations in the seat of attention with neural, cognitive, and emotional variables opens up many new paths for future research. Self-regulation of the seat of attention may be foundational for the study of previously unexamined components of a variety of cognitive states, including complex and performance-enhancing states, improved access to a variety of valuable emotional and cognitive resources and clarified understandings of the relationship between attention and meditative states. This makes attentional stances worthy of consideration for use in educational, therapeutic, self-care, and self-development contexts, and efforts should be made to replicate and extend the current findings.

Strengths of the study are the moderate to strong within-participant inter-run correlations for each attentional stance, especially in β and γ frequency ranges, as well as the robust effect size (1.06) and significance (up to *p* < 0.0001) for correlations between patterns of scalp activation associated with specific attentional stances and those associated with positive emotions in a different study (Hu et al., 2017). The main limitation is the study's small sample size, which is appropriate for an initial study on a novel cognitive aspect; results cannot be generalized to all populations until additional research is done with a larger participant sample.

## Data Availability Statement

The raw data supporting the conclusions of this article will be made available by the authors, without undue reservation.

## Ethics Statement

The studies involving human participants were reviewed and approved by Human Research Review Committee, California Institute of Integral Studies. The patients/participants provided their written informed consent to participate in this study.

## Author Contributions

GH, LL, and CT contributed to the conception and design of the study. LL and CT performed the statistical analysis and wrote the sections of the manuscript. GH wrote the first draft of the manuscript. All authors contributed to the manuscript revision, read and approved the submitted version.

## Funding

This work was funded from NSF/SL-CN 1640914 grant to LL.

## Conflict of Interest

The authors declare that the research was conducted in the absence of any commercial or financial relationships that could be construed as a potential conflict of interest.

## Publisher's Note

All claims expressed in this article are solely those of the authors and do not necessarily represent those of their affiliated organizations, or those of the publisher, the editors and the reviewers. Any product that may be evaluated in this article, or claim that may be made by its manufacturer, is not guaranteed or endorsed by the publisher.

## References

[B1] Adam H. Obodaru O. Galinsky A. D. (2015). Who you are is where you are: antecedents and consequences of locating the self in the brain or the heart. Organ. Behav. Hum. Decis. Process. 128, 74–83. 10.1016/j.obhdp.2015.03.004

[B2] Aihua L. Lin C. Xiang W. (2020). On the ways of Tai Chi regulating the body's Jing, Qi and Shen. Front. Sport Res. 2, 6–9. 10.25236/FSR.2020.020302

[B3] Ainsworth B. Eddershaw R. Meron D. Baldwin D. S. Garner M. (2013). The effect of focused attention and open monitoring meditation on attention network function in healthy volunteers. Psychiatry Res. 210, 1226–1231. 10.1016/j.psychres.2013.09.002 24135553

[B4] Alsmith A. J. Ferrè E. R. Longo M. R. (2017). Dissociating contributions of head and torso to spatial reference frames: the misalignment paradigm. Conscious. Cogn. 53, 105–114. 10.1016/j.concog.2017.06.005 28654839

[B5] Alsmith A. J. Longo M. R. (2014). Where exactly am I? Self-location judgements distribute between head and torso. Conscious. Cogn. 24, 70–74. 10.1016/j.concog.2013.12.005 24457520

[B6] Amihai I. Kozhevnikov M. (2014). Arousal vs. relaxation: a comparison of the neurophysiological and cognitive correlates of Vajrayana and Theravada meditative practices. PLoS ONE 9, e102990. 10.1371/journal.pone.0102990 25051268 PMC4106862

[B7] Amihai I. Kozhevnikov M. (2015). The influence of Buddhist meditation traditions on the autonomic system and attention. Biomed. Res. Int. 2015:731579. 10.1155/2015/731579 26146629 PMC4471252

[B8] Anglin S. M. (2014). I think, therefore I am? Examining conceptions of the self, soul, and mind. Conscious. Cogn. 29, 105–116. 10.1016/j.concog.2014.08.014 25282302

[B9] Ataria Y. Dor-Ziderman Y. Berkovich-Ohana A. (2015). How does it feel to lack a sense of boundaries? A case study of a long-term mindfulness meditator. Conscious. Cogn. 37, 133–147. 10.1016/j.concog.2015.09.002 26379087

[B10] Barbeito R. Ono H. (1979). Four methods of locating the egocenter: a comparison of their predictive validities and reliabilities. Behav. Res. Methods Instrum. Comput. 11, 31–36. 10.3758/BF03205428

[B11] Bertossa F. Besa M. Ferrari R. Ferri F. (2008). Point zero: a phenomenological inquiry into the seat of consciousness. Percept. Motor Skills 107, 323–335. 10.2466/pms.107.2.323-335 19093595

[B12] Blanke O. (2012). Multisensory brain mechanisms of bodily self-consciousness. Nat. Rev. Neurosci. 13, 556–571. 10.1038/nrn3292 22805909

[B13] Blumenfeld W. (1937). The relationship between the optical and haptic construction of space. Acta Psychol. 2, 125–174. 10.1016/S0001-6918(37)90011-8

[B14] Bottini G. Karnath H.-O. Vallar G. Sterzi R. Frith C. D. Frackowiak R. J.. (2001). Cerebral representations for egocentric space: functional-anatomical evidence from caloric vestibular stimulation and neck vibration. Brain 124, 1182–1196. 10.1093/brain/124.6.1182 11353734

[B15] Canzoneri E. Ubaldi S. Rastelli V. Finisguerra A. Bassolino M. Serino A. (2013). Tool-use reshapes the boundaries of body and peripersonal space representations. Exp. Brain Res. 228, 25–42. 10.1007/s00221-013-3532-2 23640106

[B16] Cibotaru V. (2021). The spiritual features of the experience of Qi in Chinese Martial Arts. Religions 12, 836. 10.3390/rel12100836

[B17] Cohen J. (1992). A power primer. Psychol. Bull. 112, 155–159. 10.1037/0033-2909.112.1.155 19565683

[B18] Cox P. H. (1999). An initial investigation of the auditory egocenter: evidence for a “cyclopean ear” (dissertation). North Carolina State University, Raleigh, NC, United States.

[B19] Crick F. Koch C. (2000). The unconscious homunculus. Neuropsychoanalysis 2, 3–11, 10.1080/15294145.2000.10773273

[B20] Csikszentmihalyi M. Csikszentmihalyi M. (1990). Flow: The Psychology of Optimal Experience. New York, NY: Harper and Row.

[B21] Damasio A. R. (1999). The Feeling of What Happens: Body and Emotion in the Making of Consciousness. San Diego, CA: Harcourt Brace.

[B22] Dambrun M. (2016). When the dissolution of perceived body boundaries elicits happiness: the effect of selflessness induced by a body scan meditation. Conscious. Cogn. 46, 89–98. 10.1016/j.concog.2016.09.013 27684609

[B23] Dambrun M. Berniard A. Didelot T. Chaulet M. Droit-Volet S. Corman M.. (2019). Unified consciousness and the effect of body scan meditation on happiness: alteration of inner-body experience and feeling of harmony as central processes. Mindfulness 10, 1530–1544. 10.1007/s12671-019-01104-y

[B24] De Ridder D. Van Laere K. Dupont P. Menovsky T. Van de Heyning P. (2007). Visualizing out-of-body experience in the brain. N. Engl. J. Med. 357, 1829–1833. 10.1056/NEJMoa070010 17978291

[B25] Dengis C. A. Simpson T. L. Steinbach M. J. Ono H. (1998). The cyclops effect in adults: sighting without visual feedback. Vis. Res. 38, 327–331. 10.1016/S0042-6989(97)00157-0 9536358

[B26] Dor-Ziderman Y. Ataria Y. Fulder S. Goldstein A. Berkovich-Ohana A. (2016). Self-specific processing in the meditating brain: a MEG neurophenomenology study. Neurosci. Conscious. 2016:niw019. 10.1093/nc/niw019 30397512 PMC6210398

[B27] Fetterman A. K. Juhl J. Meier B. P. Abeyta A. Routledge C. Robinson M. D. (2020). The path to God is through the heart: metaphoric self-location as a predictor of religiosity. Self Identity 19, 650–672. 10.1080/15298868.2019.1651389

[B28] Fetterman A. K. Robinson M. D. (2013). Do you use your head or follow your heart? Self-location predicts personality, emotion, decision making, and performance. J. Pers. Soc. Psychol. 105, 316–334. 10.1037/a0033374 23773045 PMC3722275

[B29] Fry G. A. (1950). Visual perception of space. Optometry Vis. Sci. 27, 531–553. 10.1097/00006324-195011000-00001 14789950

[B30] Goleman D. (1972). The Buddha on meditation and states of consciousness, Part I: the teachings. J. Transpers. Psychol. 4, 1–44.

[B31] Hanley A. W. Dambrun M. Garland E. L. (2020). Effects of mindfulness meditation on self-transcendent states: perceived body boundaries and spatial frames of reference. Mindfulness 11, 1194–1203. 10.1007/s12671-020-01330-9 33747250 PMC7968136

[B32] Hartelius G. (2007). Quantitative somatic phenomenology: toward an epistemology of subjective experience. J. Conscious. Stud. 14, 24–56.

[B33] Hartelius G. (2015). Body maps of attention: phenomenal markers for two varieties of mindfulness. Mindfulness 6, 1271–1281. 10.1007/s12671-015-0391-x

[B34] Hartelius G. Goleman J. (2016). “Body felt imagery: thoughts of the radically embodied mind,” in Transformative Imagery: Cultivating the Imagination for Healing, Change, and Growth, ed Davenport L. (London: Jessica Kingsley), 162–173.

[B35] Hering E. (1942). Spatial Sense and Movements of the Eye. Baltimore, MD: American Academy of Optometry.

[B36] Hinterberger T. Zlabinger M. Blaser K. (2014). Neurophysiological correlates of various mental perspectives. Front. Hum. Neurosci. 8, 637. 10.3389/fnhum.2014.00637 25191253 PMC4140388

[B37] Howard I. P. Templeton W. B. (1966). Human Spatial Orientation. London: John Wiley and Sons.

[B38] Hu X. Yu J. Song M. Yu C. Wang F. Sun P.. (2017). EEG correlates of ten positive emotions. Front. Hum. Neurosci. 11, 26. 10.3389/fnhum.2017.00026 28184194 PMC5266691

[B39] James W. (1890). The Principles of Psychology. New York, NY: Dover. 10.1037/10538-000

[B40] Likova L. T. (2012). “The spatiotopic “visual” cortex of the blind,” in Human Vision and Electronic Imaging XVII, Proceedings of SPIE (Burlingame, CA). 10.1117/12.912257

[B41] Likova L. T. Tyler C. W. (2008). Occipital network for figure/ground organization. Exp. Brain Res. 189, 257–267. 10.1007/s00221-008-1417-6 18604528

[B42] Limanowski J. Hecht H. (2011). Where do we stand on locating the self? Psychology 2, 312. 10.4236/psych.2011.24049

[B43] Marolt-Sender M. (2014). A phenomenological inquiry into the attention postures of flow-like states (dissertation). Sofia University, Sofia, Bulgaria.

[B44] Morsella E. Godwin C. A. Jantz T. K. Krieger S. C. Gazzaley A. (2016). Homing in on consciousness in the nervous system: an action-based synthesis. Behav. Brain Sci. 39, e168. 10.1017/S0140525X15000643 26096599

[B45] Moseley G. L. Olthof N. Venema A. Don S. Wijers M. Gallace A.. (2008). Psychologically induced cooling of a specific body part caused by the illusory ownership of an artificial counterpart. Proc. Natl. Acad. Sci. U.S.A. 105, 13169–13173. 10.1073/pnas.0803768105 18725630 PMC2529116

[B46] Nave O. Trautwein F. M. Ataria Y. Dor-Ziderman Y. Schweitzer Y. Fulder S.. (2021). Self-boundary dissolution in meditation: a phenomenological investigation. Brain Sci. 11, 819. 10.3390/brainsci11060819 34205621 PMC8235013

[B47] Neelon M. F. Brungart D. S. Simpson B. D. (2004). The isoazimuthal perception of sounds across distance: a preliminary investigation into the location of the audio egocenter. J. Neurosci. 24, 7640–7647. 10.1523/JNEUROSCI.0737-04.2004 15342730 PMC6729631

[B48] Newport R. Rabb B. Jackson S. R. (2002). Noninformative vision improves haptic spatial perception. Curr. Biol. 12, 1661–1664. 10.1016/S0960-9822(02)01178-8 12361568

[B49] Oyserman D. Elmore K. Smith G. (2012). “Self, self-concept, and identity,” in Handbook of Self and Identity, eds Leary M. R. Tangney J. P. (Guilford Press), 69–104.

[B50] Padula W. V. Subramanian P. Spurling A. Jenness J. (2015). Risk of fall (RoF) intervention by affecting visual egocenter through gait analysis and yoked prisms. Neurorehabilitation 37, 305–314. 10.3233/NRE-151263 26484522

[B51] Paillard J. (1987). “Cognitive versus sensorimotor encoding of spatial information,” in Cognitive Processes and Spatial Orientation in Animal and Man, eds Ellen P. Thinus-Blanc C. (Dordrecht: Springer), 43–47. 10.1007/978-94-009-3533-4_5

[B52] Panksepp J. (2000). The cradle of consciousness: a periconscious emotional homunculus? Commentary by Jaak Panksepp (Bowling Green). Neuropsychoanalysis 2, 24–32. 10.1080/15294145.2000.10773278

[B53] Panksepp J. (2005). On the embodied neural nature of core emotional affects. J. Conscious. Stud. 12, 158–184.

[B54] Panksepp J. Northoff G. (2009). The trans-species core SELF: the emergence of active cultural and neuro-ecological agents through self-related processing within subcortical-cortical midline networks. Conscious. Cogn. 18, 193–215. 10.1016/j.concog.2008.03.002 18485741

[B55] Roelofs C. O. (1959). Considerations on the visual egocentre. Acta Psychol. 16, 226–234. 10.1016/0001-6918(59)90096-4

[B56] Roux F. E. Djidjeli I. Durand J. B. (2018). Functional architecture of the somatosensory homunculus detected by electrostimulation. J. Physiol. 596, 941–956. 10.1113/JP275243 29285773 PMC5830421

[B57] Schäfer S. Wentura D. Pauly M. Frings C. (2019). The natural egocenter: an experimental account of locating the self. Conscious. Cogn. 74, 102775. 10.1016/j.concog.2019.102775 31279130

[B58] Serino A. Canzoneri E. Marzolla M. Di Pellegrino G. Magosso E. (2015). Extending peripersonal space representation without tool-use: evidence from a combined behavioral-computational approach. Front. Behav. Neurosci. 9, 4. 10.3389/fnbeh.2015.00004 25698947 PMC4313698

[B59] Shimono K. Higashiyama A. Tam W. J. (2001). Location of the egocenter in kinesthetic space. J. Exp. Psychol. Hum. Percept. Perform. 27, 848–861. 10.1037/0096-1523.27.4.848 11518148

[B60] Solms M. (2021). The Hidden Spring: A Journey to the Source of Consciousness. New York, NY: W. W. Norton. 10.53765/20512201.28.11.153

[B61] Solms M. Friston K. (2018). How and why consciousness arises: some considerations from physics and physiology. J. Conscious. Stud. 25, 202–238.

[B62] Starmans C. Bloom P. (2012). Windows to the soul: children and adults see the eyes as the location of the self. Cognition 123, 313–318. 10.1016/j.cognition.2012.02.002 22382132

[B63] Sukemiya H. Nakamizo S. Ono H. (2008). Location of the auditory egocentre in the blind and normally sighted. Perception 37, 1587–1595. 10.1068/p5949 19065860

[B64] Travis F. Shear J. (2010). Focused attention, open monitoring and automatic self-transcending: categories to organize meditations from Vedic, Buddhist and Chinese traditions. Conscious. Cogn. 19, 1110–1118. 10.1016/j.concog.2010.01.007 20167507

[B65] Tyler C. W. (2021). The interstitial pathways as the substrate of consciousness: a new synthesis. Entropy 23, 1443. 10.3390/e23111443 34828141 PMC8623371

[B66] van der Veer A. H. Alsmith A. J. Longo M. R. Wong H. Y. Mohler B. J. (2018). Where am I in virtual reality? PLoS ONE 13, e0204358. 10.1371/journal.pone.0204358 30304008 PMC6179224

[B67] van der Veer A. H. Longo M. R. Alsmith A. J. Wong H. Y. Mohler B. J. (2019). Self and body part localization in virtual reality: comparing a headset and a large-screen immersive display. Front. Robot. AI 6, 33. 10.3389/frobt.2019.00033 33501049 PMC7805778

[B68] Velzeboer C. M. J. (1957). Egocentral and Reciprocal Localization in Squint. Dico.25866928

[B69] Wells W. C. (1792). An Essay Upon Single Vision With Two Eyes; Together With Experiments and Observations on Several Other Subjects in Optics. London: T. Cadell.

